# First Clinical Report on Efficacy of Alternative European Viper Antivenoms in Treatment of *Vipera ammodytes* Envenomation in Croatia

**DOI:** 10.3390/toxins18040178

**Published:** 2026-04-07

**Authors:** Mihaela Čikeš Šimunković, Adrijana Leonardi, Igor Križaj, Svjetlana Karabuva

**Affiliations:** 1 Department of Infectious Diseases, University Hospital of Split, Šoltanska 1, HR-21000 Split, Croatia; mihaela.cikes@kbsplit.hr; 2 Department of Molecular and Biomedical Sciences, Jožef Stefan Institute, Jamova 39, SI-1000 Ljubljana, Slovenia; adrijana.leonardi@ijs.si (A.L.); igor.krizaj@ijs.si (I.K.); 3 University of Split School of Medicine, Department of Infectious Diseases, Šoltanska 2A, HR-21000 Split, Croatia

**Keywords:** snakebite, envenomation, *Vipera ammodytes*, antivenom, Croatia, Split

## Abstract

In Croatia, the European Viper Venom Antiserum^®^, produced by the Institute of Immunology Zagreb, was the only antiserum used to treat *Vipera ammodytes* envenomation. When production of the Zagreb antivenom ceased, three other antivenoms, Viperfav^®^, BulBio^®^, and Viekvin^®^, replaced it in clinical practice at the Department of Infectious Diseases, University Hospital Split. This study includes 34 patients envenomed by *Vipera ammodytes* during the period between 2020 and 2025: 24 (71%) suffered grade 2a envenomation, nine (26%) grade 2b, and one grade 3 (severe envenomation). None were admitted to the Intensive Care Unit. All patients received antivenom: 16 received Viperfav^®^, 17 BulBio^®^, and one Viekvin^®^. All grade 2a patients were treated with a single dose of antivenom. Among grade 2b patients, four received one dose and two received two doses of Viperfav^®^, while one received one dose and two received two doses of BulBio^®^. The grade 3 patient received two doses of BulBio^®^. In all cases, treatment was successful and patients were discharged from hospital after an average of 3.97 days. Patients with pronounced neurotoxic signs did not require treatment with multiple doses of antivenom. All antivenoms proved effective. No adverse reactions or fatalities were observed.

## 1. Introduction

Ophidism refers to envenomation by snake venom. Globally, ophidism affects 3 million people annually, resulting in about 125,000 deaths [1,2,3,4]. In Europe, approximately 7500 snakebites are recorded each year, with 1000 cases of severe envenomation and 4 fatal outcomes [1,2,3,4]. Venomous snakebites in Europe are mainly caused by *Viperidae* species of the *Vipera* genus. The main *Vipera* species of medical importance in Europe are *Vipera ammodytes*, *V. aspis*, *V. berus*, *V. latastei*, *V. seoanei* and *V. ursinii* [5]. The mortality rate in Europe caused by *Vipera* snake envenomation ranges from 0.3% to 5%, and in Croatia from 0.4% to 1.8% [1,6]. Three venomous snakes are found in Croatia: the nose-horned viper (*V. ammodytes*), the common adder (*V. berus*), and the meadow viper (*V. ursinii*). Clinically, the most severe envenomations are inflicted by *V. ammodytes*, the largest European snake. The venom of *V. ammodytes* is well characterised and is particularly known for its potent haemorrhagic and neurotoxic effects [7,8,9].

Generally, data on snakebites are often unreliable due to insufficient interest, as shown by the lack of mandatory reporting and standardisation of notifications by health services. However, records of ophidism in Croatia, both in adults and especially in children, have been very well documented since 1968 ([6,10] Lukšić, personal communication). In Croatia, the University Hospital Centre Split (UHC Split) has the most clinical experience in this area. Currently, no standardized protocol exists for viper envenomation treatment across Europe. Treatment varies between countries depending on the clinical experience of hospital staff. It has, however, been recommended that moderate and severe clinical viper envenomations are treated with antivenom [5]. All data on ophidism in Croatia from 1968 to 2020 have thoroughly described the clinical presentation and treatment measures ([6,10] Lukšić, personal communication). During this period, our clinical practice exclusively used the European Viper Venom Antiserum^®^ (Zagreb, Croatia), produced by the Institute of Immunology Zagreb, until its application was stopped in 2020. In Southeast Europe, the availability of specific antivenom for *V. ammodytes* venom is crucial. ViperaTab^®^ (Newcastle Emlyn, United Kingdom) antivenom, developed for *V. berus* venom, is not sufficiently effective in treating severe cases of *V. ammodytes* envenomation [11]. Therefore, since 2021, UHC Split has used other commercially available antivenoms, Viperfav^®^ (Lyon, France), BulBio^®^ (Sophia, Bulgaria) and Viekvin^®^ (Belgrade, Serbia), which are also effective against *V. ammodytes*.

The primary goal of this study is to describe, for the first time, the detailed clinical and therapeutic experiences of using three different anti-*V. ammodytes* sera as alternatives to the serum produced at the Institute of Immunology in Zagreb for treating *V. ammodytes* venomous bites. The study also aimed to highlight epidemiological and clinical characteristics, laboratory findings, and differences among various antivenoms and supportive therapies used to treat envenomed patients to develop national guidelines for the management of clinical cases of ophidism in Croatia.

## 2. Results

### 2.1. General and Demographic Results

**Figure 1:**
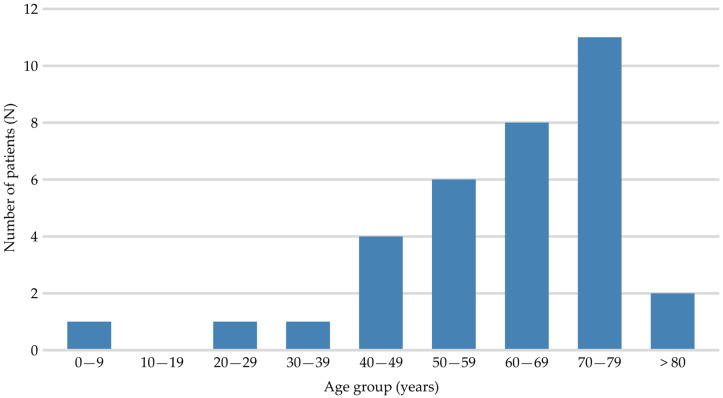
Age distribution of patients (N = 34) bitten by *V. ammodytes*.

During the study period, 34 patients envenomed by *V. ammodytes* were hospitalised in the Department of Infectious Diseases at UHC Split. There were 20 men (59%) and 14 women (41%). The oldest patient was an 84-year-old man, and the youngest was a four-year-old boy. The median age was 64.5 years. The age distribution of the patients is shown in Figure 1. Most snakebite victims (29/34) were between 40 and 79 years of age (Figure 1).

### 2.2. Epidemiological Data

**Figure 2:**
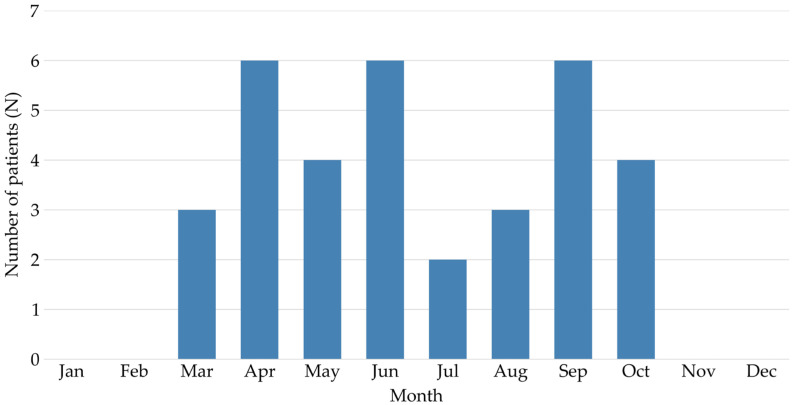
Monthly distribution of *V. ammodytes* bites (N = 34).

The distribution of *V. ammodytes* bites by month is shown in Figure 2. Most snakebites were recorded from spring to late summer, from the end of March to the end of September, with peaks (N = 6) in April, June, and September.

The average time of day when bites occurred was 13:31, with the earliest at 8:00 and the latest at 19:30. These times were expected due to human outdoor activities during the day and the circadian activity of the *V. ammodytes* [12]. Among the patients, 27 (79%) were local residents and seven (21%) were non-residents, either tourists or people not living in Croatia but with relatives or family there. The circumstances of the snakebites were as follows: housework (24/34), leisure activities (9/34), and playing outdoors (1/34). According to the reported habits, seven out of 34 patients (21%) were consuming cigarettes and alcohol.

### 2.3. Vital Parameters and Clinical Records on Admission to the ED

All vital parameters and a summary of the clinical records of study patients (N = 34) on admission to the ED are shown in Table 1. All patients were afebrile, and most were haemodynamically stable (Table 1). Four patients were hypotensive, eight had tachycardia, and one was in shock, presenting as somnolent and dyspnoeic (Table 1).

The bite was mainly located on the upper extremity (23/34; 68%). The distribution of bites in the upper and lower limbs did not differ between men and women. No bites on the head and torso were registered. In total, 15 patients (44%) had at least one underlying chronic disease, but none were immunosuppressed (Table 1). According to the severity of envenomation, 24 patients (71%) were scored as grade 2a, nine (26%) as grade 2b, and only one patient as grade 3. None of the study patients were admitted to the ICU. During the study period, there were no patients classified as grade 0 or 1.

The median time from snakebite to arrival at the ED was 168 min (Table 1). The maximum time from snakebite to arrival at the ED was 1140 min (19 h) in the case of a patient who received on-site treatment from a medical doctor in the emergency team but refused transfer to hospital. After observing progression of the local symptoms, the family brought the patient to the ED, and the patient was hospitalised.

The median time from snakebite to antivenom administration was 183 min. The minimum time was 20 min, in the case of a patient who was in shock and received one dose of antivenom (IM) immediately in the field.

None of the observed parameters in Table 1 differ among the three used antivenoms.

The routine practice at UHC Split is to administer antivenom immediately after physical examination and grading of envenomation. All patients received antivenom within 30 min of arrival at the hospital.

### 2.4. Local and Systemic Features and Complications of Envenomation

Local and systemic symptoms and signs of envenomation are presented in Table 2. All patients experienced pain after *V. ammodytes* bite and developed oedema, while 62% had redness at the bite site and 91% developed a local haematoma. The most common systemic manifestations of envenomation were vomiting (32%) and diarrhoea (9%).

A haemorrhagic blister was the only local complication recorded, occurring in one patient who sustained a double bite from *V. ammodytes* on a finger. In total two patients suffered multiple bites (both on arm fingers).

A total of 24 patients (71%) experienced some form of observed systemic complication (Table 2). Laboratory-confirmed coagulation disorder was the most common systemic complication recorded in our study patients (16/34), but none of these cases had clinically manifested bleeding. Thrombocytopenia was observed in nine cases and leukocytosis in eight.

One of the most dramatic and urgent clinical conditions observed in eight patients was paresis or paralysis of the cranial nerves (Table 2). The most common cranial nerve paresis involved the oculomotor nerve, clinically manifested as bilateral eyelid ptosis, and was recorded in all eight patients. Other signs of neurological impairment included one patient who developed ophthalmoplegia, one with dysphagia, and one with dysphonia. No cases of paranesthesia, lip paralysis, or muscle weakness were observed. Typically, eyelid ptosis appeared two to four hours after the bite and lasted for about 72 h, after which it usually resolved completely. Subjects with other signs of neurotoxicity generally recovered fully after 72–96 h. No neurological sequelae were present at discharge. One patient experienced a disorder of consciousness while in shock. Rhabdomyolysis and/or myositis was observed in six patients, but all fully recovered within 24–48 h. None of the patients developed acute kidney disorder, acute liver damage, acute myocardial injury, or acute respiratory failure, and anaemia was not observed in any case.

None of the observed clinical data in Table 2 differ among the used antivenoms.

### 2.5. Laboratory Parameters

All laboratory parameters of the study patients are presented in Table 3.

Leukocytosis was observed in eight patients, and the clinical impression was that the level of leucocytosis was higher the later the patients arrived at the hospital and the more time had elapsed since the snakebite; the same observation applied to the neutrophil count. Red blood cell parameters were within the reference intervals for both men and women. The average Ptc was 210 × 10^9^/L, with a range of 48–340 (minimum–maximum). CRP values were predictably low and had no clinical significance in assessing the severity of envenomation in these patients. No patient had an estimated glomerular filtration rate lower than predicted for their age. No clinically significant electrolyte disturbances were observed, except for the expected hypokalaemia in patients who vomited or had diarrhoea during systemic signs of envenomation. Liver enzyme levels were within reference ranges, except in patients with a history of chronic alcohol consumption, who, in addition to elevated liver enzymes, also had elevated GGT. CK and LDH levels increased proportionally in cases of rhabdomyolysis and/or myositis, as clinically expected. Although several patients had elevated hs-TnT above the strict reference interval, not a single patient presented with a clinical picture of an acute cardiac incident—none developed chest pain, ECG changes, or showed any dynamics of hs-TnT increase. Notably, 16 out of 34 patients had laboratory-confirmed coagulation disorders (Table 2), but no thrombosis or signs of bleeding were clinically recorded. Detailed coagulation characteristics of the study patients are shown in Table 3.

### 2.6. Treatment Data and Clinical Outcomes

Provided treatment data are presented in Table 4.

Pre-hospital treatment by on-site medical emergency assistance included limb immobilisation (3/34; 9%), administration of antivenom in the field (3/34; 9%), and provision of supportive therapy during transport to the hospital facility (24/34; 71%). Of the three patients who received antivenom, one was in a state of shock, received one dose IM within 20 min of the *V. ammodytes* bite, and was immediately transferred to the ED by helicopter emergency medical service. The other two patients were classified on-site as grade 2b by an emergency medicine specialist and each received one dose of antivenom IM. An additional dose was deemed necessary in hospital. All other study patients (31/34) also received antivenom in hospital.

Three different antivenoms and routes of administration were used to treat envenomations. Viperfav^®^ was administered IV to 16 patients, BulBio^®^ was given to 17 patients (eight received it IM and nine both IM and SC), and Viekvin^®^ was administered to one patient via the IM route. In total, 29 patients (85%) were treated with a single dose of antivenom, while five patients (15%) received two doses. In all five cases requiring a double dose due to the severity of envenomation—grade 3 (one case of shock) and grade 2b (four cases)—the main reason for multiple doses was the progression of neurotoxicity. No patient required a third dose of antivenom.

Our patients received the following types of supportive therapy (Table 4): antibiotics (18/34; 53%), human anti-tetanus immunoglobulins (33/34; 97%), tetanus vaccine—tetanus toxoid (33/34; 97%), antihistamines (31/34; 91%), corticosteroids (31/34; 91%), and oxygen therapy (only one patient, who was in shock). None of the subjects received platelet and/or erythrocyte transfusions, anticoagulants, vasopressors, or mechanical ventilation. Only one patient underwent surgical intervention—incision of a haemorrhagic blister—while fasciotomy, necrectomy, and limb amputation were not performed in any case in this study.

Clinical outcomes in our patients were favourable; the average length of hospital stay for all grades of envenomation was 3.97 days, with no fatalities (Table 4).

The comparison between the grade of envenomation and the number of antivenom doses is shown in Table 4. All patients with grade 2a envenomation (N = 24) were treated with a single dose of antiviper serum. Among the nine patients with grade 2b envenomation, five received one dose and four received two doses—Viperfav^®^ IV and BulBio^®^ IM, respectively. One patient with grade 3 envenomation status received two doses of BulBio^®^ IM and recovered well.

## 3. Discussion

In Split–Dalmatia County and Croatia in general, venomous snakes are active from early spring to late autumn, with most snakebite envenomations occurring when people spend time in nature. The increasing number of snakebites in Croatia is attributed to the growing number of trips and visiting tourists, especially during the warmer months. In our study, most of *V. ammodytes* venomous bites were recorded between the end of March and the end of September, similar to other European countries [1,13,14,15]. Seventy-nine percent of snakebite victims requiring hospital care were local residents, while the remainder were non-residents, which is notably high. All 25 local residents were bitten during work or other outdoor activities, whereas all nine non-residents encountered snakes during leisure activities. Our epidemiological data correspond to those previously described [1,6]. This study also recorded a higher incidence of snakebites in men (59%) than in women (41%), which matches observations from earlier studies in Europe [1] and Croatia [6,10].

Our patients were usually bitten by the snake on one of their upper limbs (68%). Bites to the upper extremity were also most frequently recorded in previous studies [1,6,10,15]. However, some other studies report viper bites more often on the lower extremity [16,17,18], presumably due to the specific activities of the victims at the time of the snake bite. Nevertheless, this did not result in any clinically significant differences. In our study, we paid attention to the chronic diseases from which the victims suffered (15/34). As snake envenomation is an acute event, its impact on chronic diseases has not been studied so far. Among the chronic diseases, cardiovascular diseases (11/34) and diabetes (5/34) were most common in our patients. This proportion of chronic diseases was expected given the age of the subjects included in our study. Clinically, envenomation and/or antiserum treatment did not appear to influence the course of these chronic diseases. However, to draw firm conclusions, follow-up data for these patients would be necessary to observe possible late side effects of envenomation and antiserum therapy. None of our study patients developed adverse reactions after receiving antivenom therapy. The prevalence of early hypersensitivity reactions to snake antivenom in general, not only to antiviper sera, is very frequent (up to 40%), but mainly, the reactions are not severe [2,5,11]. Late hypersensitivity reactions that occur due to the deposition of excessive circulating immune complexes in patients treated with antivenin for snakebites has been frequently reported in the USA, but not in Europe, nor in Croatia [2,5,6,10,11].

In Croatia, patients with envenomation grades 0 and 1 are usually not hospitalized, except in the case of small children [10] and/or under specific paramedical circumstances, such as when the patient’s residence is far from the hospital and/or when it is difficult to perform check-ups outside the care facility. As mentioned, in our study there were no hospitalized patients with mild envenomation or dry bite. Therefore, data on these cases are lacking in our study. Other European countries, such as Slovenia, France, Italy, and Turkey, use different snakebite reporting systems, which also include such cases [14,15,17,19,20,21]. In our study, most cases were graded 2a (71%) and 2b (26%) with only one case graded 3. Previous studies from Croatia [6,10] reported the severity of snakebites in patients using the old classification system [22,23,24]. According to these studies, 15.1%, 40.5%, 26.0%, and 18.0% of *V. ammodytes* envenomations in adults were classified as minor, mild, moderate and severe, respectively [6]. Two envenomations were fatal (0.4%) [6]. In children, 9.4%, 35.0%, 30.6%, and 24.4% of envenomations were minor, mild, moderate and severe, respectively [10]. One envenomation (0.6%) unfortunately ended fatally [10]. These data may seem incomparable with our present results, but in addition to the different envenomation severity classifications used, it should be noted that indications for hospital treatment have changed significantly in recent years and that the outpatient care service, which reduces the number of hospitalisations, is expanding.

In our study, the median time from the snakebite to arrival at the ED was 168 min, and from snakebite to administration of antivenom was 183 min. This shows that patients reach hospital quickly and receive targeted therapy promptly. Studies from Slovenia report similar times [8,15]. Due to relatively high awareness of the dangers posed by snakebites, most patients in Split-Dalmatia County reported the incident to hospital staff as soon as possible. Rapid emergency medical intervention and/or patient arrival at the ED is therefore not surprising. Very few clinical studies present the time from snakebite to hospital admission, and the time from snakebite to receiving viper antivenom [11,15]. In Croatia, there is currently no national guideline for the treatment of snakebite or the use of antivenom in the field. However, an informal rule exists that in cases of (pre)shock condition of the snakebite victim, administration of antivenom in the field is obligatory. It is concerning that in no more than three patients (out of 34) was the bitten extremity immobilised upon arrival at hospital. Clearly, first aid after viper envenomation in the field, including limb immobilisation, should be further promoted among non-professionals. There are no published data on this issue in other European countries. Experts no longer recommend traditional first aid approaches, as most or all have been shown to be ineffective. Supportive therapy in the field is common practice in Croatia, but it is presented here for the first time. In our study, 71% (24/34) of bitten individuals received IV infusion of 0.9% NaCl solution, IM injection of antihistamines, and IV or IM injection of corticosteroids to prevent possible complications after antiserum injection.

Local symptoms and signs of envenomation by *V. ammodytes* venom, as well as systemic manifestations in our patients, are consistent with previous clinical reports [15]. Pain and oedema were present in all patients, while haematoma occurred in 91% and vomiting in 32% of cases. Only one patient was in a state of shock and exhibited changes in mental status. Previous studies from Croatia report the occurrence of haemorrhagic blisters in 13% of the study population and in 20% of children [6,10]. However, in the present study, only one patient developed a haemorrhagic blister on the finger, probably due to a double bite at the same site.

Systemic complications after envenomation were reported in 71% of our patients (24/34). Paresis or paralysis of cranial nerves was most prominent, typically presenting as bilateral eyelid ptosis, ophthalmoplegia, dysphagia, or dysphonia. Neurotoxicity is well documented in many studies describing *V. ammodytes* envenomation in both preclinical and clinical studies [6,8,10,20,21,25,26]. In our study, neither paraesthesia and lip paralysis nor muscle weakness were observed. Previous case reports and pharmacokinetic evaluations have indicated that a single dose of ViperFav^®^ may not be sufficient for treating serious cases of *V. ammodytes* envenomation, primarily due to ineffectiveness against neurotoxic venom components [15]. Nevertheless, further studies have demonstrated the clinical efficacy of ViperFav^®^ in such cases [27]. Our patients with pronounced neurotoxic signs also did not require treatment with multiple doses of antivenom.

The laboratory parameters of our patients were not significantly or acutely altered by *V. ammodytes* envenomation, except for those reflecting the observed disorders: blood coagulation changes, thrombocytopenia (9/34), leukocytosis (8/34), and myositis (6/34). These findings are consistent with the multicentre study of snakebites in Central and Southeastern Europe [15]. The effects of *V. ammodytes* venom and its toxins on haemostasis are well known [27,28]. Interestingly, although 16 of the 34 patients in our study had a laboratory-confirmed coagulation disorder following toxic *V. ammodytes* bite, none developed clinical manifestations of coagulopathy. In contrast, a case report from Italy described a patient who developed coronary artery thrombosis after a *V. aspis* bite despite chronic dual antiplatelet therapy with ticagrelor and acetylsalicylic acid [28]. Clinical cases of *V. ammodytes* envenomation in Croatia report thrombocytopenic purpura and thrombocytopenia, confirming the regular manifestation of complex coagulation disorders in victims [29]. Notably, in our study, none of the patients developed acute myocardial injury, despite the cardiotoxicity of *V. ammodytes* venom having been demonstrated in rats [30].

Our patients with grade 2a envenomation (N = 24) appeared to be equally effectively treated with a single dose of either ViperFav^®^, BulBio^®^, or Viekvin^®^ (with the note that only one patient was treated with Viekvin^®^ during this study period). No adverse reactions were observed. The question remains whether grade 2a cases require the use of antiviper serum at all. Of the nine grade 2b patients, five were successfully treated with only one dose of antiviper serum, ViperFav^®^ IV (four patients) and BulBio^®^ IM (one patient). The remaining four patients received two doses of antivenom, either two doses of ViperFav^®^ IV or two doses of BulBio^®^ IM. None of these patients were haemodynamically unstable; the main reason for an additional dose of antivenom was progression of neurotoxicity. Grade 2b patients with pronounced neurotoxicity were treated equally successfully with both antiviper sera. It appears that all three antisera were similarly effective in protecting victims, as the time from the *V. ammodytes* venomous bite to antiserum administration was approximately the same. Although a standardised protocol for antivenom administration does not exist, parenterally administered polyclonal antivenoms remain the mainstay of snakebite therapy [1,25]. The widely accepted view is that the IV route should be preferred over the IM route to achieve better therapeutic efficacy. It has been demonstrated that venom neutralisation in both the systemic circulation and the lymphatic system is important for a favourable clinical outcome in severe envenomation cases [31]. The lymphatic system is a relevant body compartment through which absorption of venom components occurs [31], and it is targeted more effectively by the antiserum if administered IV. It is important to note that our patient with grade 3 envenomation, who was in shock, received serum only IM (as this was the only available option), first in the field 20 min after the snakebite and again upon arrival at the ED. His recovery was rapid. After six to eight hours, the patient became haemodynamically stable, so injection of an additional dose of antiserum was not indicated.

The manufacturer of BulBio^®^ states in its product information that in some cases of envenomation, where local symptoms predominate, in addition to the recommended IM application of serum, 1 mL of serum should be administered SC near the bite site. In our study, in some cases (9/34), patients received BulBio^®^ antiserum according to this protocol. The rationale for this practice was to accelerate the resolution of local effects; however, in our study, such an effect was not observed.

A commendable aspect of *V. ammodytes* envenomation therapy in Croatia is the significant reduction in antibiotic use. In our study, only 53% of patients received antibiotics, whereas in previous years almost all victims (97%) received antibiotic treatment [6]. The significantly lower use of antibiotics in envenomation treatment did not result in an increased number of infections; in fact, not a single patient in our study developed an infection at the bite site or showed clinical signs of systemic infection. Our experience should be incorporated into the national guidelines. In other parts of the word, in cases of viper bites and other snakebites, antibiotic therapy is also not routinely recommended [32,33,34,35].

## 4. Conclusions

In Croatia, all three different antivenoms (Viperfav^®^, BulBio^®^, Viekvin^®^)—alternatives to the European Viper Venom Antiserum^®^—proved to be effective in treating *V. ammodytes* envenomed patients. In cases of severe envenomation with pronounced neurotoxicity, there was no need to provide multiple doses of antiviper serum. The significantly lower use of antibiotics did not increase the frequency of infections nor affect the clinical outcome; therefore the new national guidelines should include a recommendation not to routinely prescribe antibiotic therapy for the treatment of *V. ammodytes* envenomation.

When using new types of antiviper sera, it is advisable to conduct pharmacological studies on the relationship between venom and antivenom proportions. As such studies are often not feasible in smaller medical centres, frequent and detailed clinical studies, case series, and reports should be published, as experiences from other research and hospitals in using diverse antiviper sera are very valuable and reliable in clinical practice.

## 5. Materials and Methods

### 5.1. Antisera

#### 5.1.1. Viperfav^®^

Viperfav^®^ was supplied by Sanofi Pasteur, Lyon, France. It is an antivenom containing equine immunoglobulin F(ab’)2 fragments which have the property of neutralising the venom of three species of vipers: *V. aspis* (≥1000 ELISA Units/4 mL dose), *V. ammodytes* (≥1000 ELISA Units/4 mL dose), and *V. berus* (≥500 ELISA Units/4 mL dose) [36]. These equine immunoglobulin F(ab’)2 fragments bind to the venom antigens present in the circulation to form inactive F(ab’)2-antigen complexes, thereby reducing the concentration of free venom in the organism. Experimental testing has shown that they are responsible for redistributing the venom antigens from peripheral tissues sites to the vascular compartment, where they are bound and inactivated. Estimated elimination half-life of equine F(ab’)2 fragments is 40 to 105 h in humans. The final product is thermostable from +2 to +8 °C in a liquid form and indicated only for IV administration [36]. F(ab’)2 immunoglobulin fragments are typically described as having a molecular mass of 100–110 kDa [37]. Fab fragments have the largest volume of distribution and readily reach extravascular compartments [31,37]. They are catabolized mainly by the kidney, having a more rapid clearance than F(ab’)2 fragments and IgG [37]. IgG molecules have a lower volume of distribution and a longer elimination half-life, showing the highest cycling through the interstitial spaces in the body [31,37]. IgG elimination occurs mainly by extrarenal pathway. F(ab’)2 fragments display a pharmacokinetic profile intermediate between those of Fab fragments and IgG molecules [37]. Such different pharmacokinetic properties have implications for the pharmacodynamics of these immunobiologicals, since a pronounced mismatch has been described between the pharmacokinetics of venoms and antivenoms [37,38]. Pharmacokinetic analysis in animal models (rabbits) showed that the plasma F(ab′)2 concentration followed a biexponential decline after intravenous bolus administration with distribution and elimination half-lives of 2.66 ± 0.18 h and 49.69 ± 4.13 h, respectively [38]. No cases of anaphylaxis or serum sickness were observed in patients treated with ViperFav^®^ in Slovenia [27].

#### 5.1.2. BulBio^®^

BulBio^®^ was from Bulbio-National Center of Infectious and Parasitic Diseases, Sophia, Bulgaria. It contains equine F(ab’)2 immunoglobulin fragments raised against *V. ammodytes* venom [39]. It is monospecific and indicated for the treatment of systemic envenomation caused by the venom of the *V. ammodytes*, but it may be used for *V. berus*, *V. aspis*, and *V. ursinii* envenomations as well. No information for its elimination half-life is available. This product is prepared in a liquid formation, thermostable from +2 to +8 °C, and may be applied IM and/or SC [39]. There are no published studies investigating this antivenom in animal models available. No human studies report cases of hypersensitivity.

#### 5.1.3. Viekvin^®^

Viekvin^®^ is manufactured by the Institute of Virology, Vaccine and Sera, TORLAK, Belgrade, Serbia. It is an antiserum containing F(ab)2 fragments of equine immunoglobulins produced by immunization using *V. ammodytes* venom. It is used for neutralization of the venoms of *V. ammodytes* and *V. berus*, while it is not effective against other European viper venoms. A total of 1 mL of Viekvin^®^ neutralizes 100 LD50 of the *V. ammodytes* venom and 50 LD50 of the *V. berus* venom [40]. There are no data for its elimination half-life. This product is made in a liquid form, thermostable from +2 to +8 °C, for parenteral administration [40]. There are no published experimental studies with this antivenom in animal models available. At this moment, no human studies report cases of hypersensitivity.

### 5.2. General Information About the Study

This retrospective study covers the period from 1 January 2021 to 30 September 2025 at the Department of Infectious Diseases at UHC Split. UHC Split, a regional hospital, is a tertiary referral centre located in Split, Split-Dalmatia County, Croatia. It serves a local population of about one million citizens of the Republic of Croatia, approximately 500,000 residents from the southern part of neighbouring Bosnia and Herzegovina, and about 500,000 tourists during the summer.

All clinical cases of ophidism in this area are traditionally treated in the Department of Infectious Diseases, except for life-threatening cases of envenomation, which, according to the doctor’s assessment, are sometimes initially admitted to the Intensive Care Unit (ICU) and then transferred to the Department of Infectious Diseases for further care.

It should be noted that the Government of the Republic of Croatia declared the COVID-19 pandemic from March 2020 to May 2023. During this period, special working conditions were in place in Croatian hospitals, and the annual number of patients hospitalised due to ophidism at UHC Split was significantly lower than usual.

This research was conducted in accordance with all applicable legal regulations, ethical principles, and guidelines (WMA Declaration of Helsinki 1964–2013, Principles of Good Clinical Practice, Charter of Human Rights in Biomedicine, European Code of Research Integrity). All necessary steps and measures were taken to protect the identity of study participants and to safeguard collected personal data in accordance with the EU General Data Protection Regulation 2016/679 (GDPR) and the Law (42/2018) governing its implementation in the Republic of Croatia. The study was approved by the Ethics Committee of UHC Split (protocol class: 520-03/26-01/18; date of approval: 18 February 2026). No patient involved in this study is identifiable in any way.

### 5.3. Patients

The patient was included in this study only if the snake involved in the incident was unequivocally identified as *V. ammodytes*, either by providing the medical staff with a photograph of the snake or by bringing a killed specimen to the Emergency Department of the Department of Infectious Diseases, UHC Split (ED). There were 34 such cases (N = 34) during the specified period.

Envenomation severity was ranked according to the Audebert classification scheme, as modified by Boels et al. [41,42], as follows:

- grade 0 (dry bite): fang marks with no oedema or local reaction;
- grade 1 (mild envenoming): local oedema around the bite area with no systemic symptoms;
- grade 2 (moderate envenoming);
- grade 2a: regional oedema (most of the bitten limb) or haematoma;
- grade 2b: grade 2a features plus moderate general symptoms: mild hypotension, vomiting, diarrhoea, neurotoxicity, or laboratory findings indicating severity: platelets < 150 × 10^9^/L, leukocytes > 15 × 10^9^/L, INR > 1.5, fibrinogen < 2 g/L;
- grade 3 (severe envenoming): extensive oedema spreading to the trunk or severe general symptoms, such as hypotension (systolic blood pressure < 80 mmHg), shock, and bleeding.

All patients were enrolled after providing their consent regarding the grade of envenomation and the decision to administer antivenom. The decision-making teams comprised three infectious disease specialists.

All data collected in this retrospective study were divided into several sections:

- general and demographic records (gender; age group [0–9 years, 10–19 years, 20–29 years, 30–39 years, 40–49 years, 50–59 years, 60–69 years, 70–79 years, >80 years]);
- epidemiological data (time of the day when the snakebite occurred [hour]; month of the snakebite; status as resident or non-resident citizen; circumstances of the snakebite: housework, leisure activities, playing outdoors; patients’ habits: cigarette and alcohol consumption);
- vital parameters on admission to the ED (temperature [°C]; pulse [beats/min]; systolic and diastolic blood pressure (RR) [mmHg]; respiratory frequency [breaths/min]; peripheral oxygen saturation [%]; state of consciousness: awake, somnolent, stupor, coma; hypotension; tachycardia; dyspnoea; chest pain; changes in the electrocardiogram (ECG));
- clinical records (location of the bite: upper, lower extremity, or other location; chronic diseases: cardiac and vascular disease, neurological diseases, pulmonary disease, liver diseases, mental illnesses, diabetes, malignant diseases, autoimmune disorders and/or immunosuppression, hypothyroidism, benign urological diseases; severity grade of envenomation; time from snakebite to ED admission [minutes]; time from snakebite to administration of antivenom [minutes]);
- local symptoms and signs of envenomation (pain, oedema, redness, haematoma, enlarged regional lymph nodes);
- systemic manifestations of envenomation (vomiting, diarrhoea, changes in mental status, state of shock);
- local complications of envenomation (haemorrhagic blister, compartment syndrome, necrosis of the skin and/or muscle, thrombosis and/or thrombophlebitis, infections);
- systemic complications of envenomation (paresis or paralysis of cranial nerves, disorders of consciousness, shock, clinically manifested bleeding disorder, laboratory-confirmed coagulation disorder, acute kidney disorder, acute liver damage, acute myocardial injury, acute respiratory failure, rhabdomyolysis and/or myositis, anaemia, thrombocytopenia, leucocytosis);
- laboratory parameters (white blood cell count (WBC) [/L], neutrophil ratio [%], red blood cell count (RBC) [/L], haematocrit (Htc) [L/L], haemoglobin (Hgb) concentration [g/L], platelet count (Ptc) [/L], C-reactive protein (CRP) concentration [mg/L], glucose concentration [mmol/L], blood urea nitrogen (BUN) concentration [mmol/L], serum creatinine concentration [µmol/L], sodium (Na) concentration [mmol/L], potassium (K) concentration [mmol/L], chloride (Cl) concentration [mmol/L], aspartate aminotransferase (AST) activity [U/L], alanine aminotransferase (ALT) activity [U/L], ɣ-glutamyl transferase (GGT) activity [U/L], total bilirubin concentration [µmol/L], lactate dehydrogenase (LDH) activity [U/L], creatine kinase (CK) activity [U/L], high sensitive-troponin T (hs-TnT) concentration [ng/L], N-terminal pro B-type natriuretic peptide (NT-proBNP) concentration [pg/mL], prothrombin time (PT) [seconds], activated partial thromboplastin time (aPTT) [seconds], D-dimer concentration [mg/L], plasma fibrinogen concentration [g/L]);
- treatment data (information about on-site assistance provided by emergency medical services: application of a tourniquet, limb immobilisation, administration of antivenom, other supportive therapy during transport to hospital; administration of antivenom in hospital; type of antivenom: Viperfav^®^, BulBio^®^, Viekvin^®^; total number of serum doses; route of antivenom administration: intravenous (IV), intramuscular (IM), subcutaneous (SC), combination of IM and SC (IM & SC); side-effects after antivenom administration: early and late; supportive therapy: antibiotics, human tetanus immunoglobulins (HTIG), tetanus vaccine—tetanus toxoid (TT), antihistamines, corticosteroids, oxygen therapy, platelet and/or erythrocyte transfusion, anticoagulants, vasopressors, mechanical ventilation; surgical intervention: incision of haemorrhagic blister, fasciotomy, necrectomy, limb amputation);
- clinical outcomes (length of hospital stay [days]; mortality).

Hypotension was defined as systolic blood pressure below 90 mmHg. Tachycardia was defined as a heart rate exceeding 100 beats/min. Dyspnoea was defined as a respiratory frequency below 20 breaths/min accompanied by peripheral oxygen saturation lower than 90%. Chest pain was defined as pain in the mediastinum lasting more than one minute and accompanied by changes in the electrocardiogram and/or a positive elevation dynamic of hs-TnT (reference interval < 14 ng/L). Changes in the electrocardiogram were defined as acute changes compared to previous ECG records or clear evidence of myocardial injury. Paresis or paralysis of cranial nerves included ptosis, ophthalmoplegia, dysphagia, dysphonia, any other type of neurological impairment, or a combination of these impairments. Disorder of consciousness was defined as somnolence, stupor, or coma. Shock was defined as prolonged hypotension (systolic blood pressure < 90 mmHg) unresponsive to intravenous fluids and accompanied by other organ failure. Clinically manifested bleeding disorder was defined as any type of evident bleeding. Laboratory-confirmed coagulation disorder was diagnosed according to abnormal levels of PT (reference interval for PT > 0.70 s) and aPTT (reference interval for aPTT 21.6–28.7 s). All coagulation values were adjusted for age and gender. None of the patients included in the study received anticoagulant therapy or any other chronic medication that could affect coagulation parameters. Acute kidney disorder was defined as at least a twofold increase in serum creatinine (reference interval 64–104 µmol/L, adjusted for age and gender) accompanied by oliguria or anuria. Acute liver damage was diagnosed when AST and ALT activity levels were at least twice the upper normal limit (reference interval: AST 11–38 U/L, ALT 12–48 U/L). Acute myocardial injury was defined as chest pain with ECG changes and a corresponding dynamic elevation of hs-TnT. Acute respiratory failure was defined as tachypnoea or dyspnoea combined with hypoxia (partial pressure of oxygen in arterial blood below 7.5 kPa). Rhabdomyolysis and/or myositis was defined by CK levels at least twice the upper normal limit (reference interval for CK: 50–177 U/L, adjusted for gender and age). Anaemia was defined as a low Hgb level (reference interval for Hgb: males 138–175 g/L, females 120–160 g/L). Thrombocytopenia and leucocytosis were defined as Ptc below 150 × 10^9^/L and leukocyte count above 11 × 10^9^/L, respectively.

### 5.4. Antivenom Therapy

At the beginning of our study, according to the hospitals’ decision, Viperfav^®^ was first available in UHC Split, followed by BulBio^®^, and currently Viekvin^®^ is used.

One package of ViperFav^®^ contains an ampoule with 4 mL of concentrate [36]. To prepare the infusion solution, the concentrate was diluted in 100 mL of 0.9% NaCl solution. The antivenom solution was administered to a patient IV over one hour.

One package of BulBio^®^ antiserum contains one ampule with 10 mL of solution for IM application and one ampule with 1 mL of solution for SC application. Patients are usually given one ampoule IM, although the manufacturer allows for the possibility of giving one ampoule IM along with one ampoule SC in the area around the bite. Thaw ampule containing 1 mL of solution may also be used for sensibilisation testing. The decision, regarding the administration route is at the clinician’s discretion [39].

One package of Viekvin^®^ serum contains one ampule of 5 mL solution for parenteral administration—usually IM, but in the case of life-threatening conditions, it must be given IV. For IV application, the 1 mL-ampule solution is diluted in 250 mL of 0.9% NaCl solution and administered over 30 min [40].

### 5.5. Statistical Analysis

Data were calculated using Microsoft Office Excel 2019 (Microsoft Corporation, Redmond, WA, USA) and the Statistical Package for Social Sciences for Windows (version 23.0, SPSS Inc., Chicago, IL, USA). Categorical variables are presented as number and percentage (N, %). Quantitative data are expressed as mean value with standard deviation, or as median, maximum, and minimum. Results are presented in tables and figures.

## Figures and Tables

**Table 1:** Vital parameters and clinical records of study patients (N = 34) at emergency admission in the Department of Infectious Diseases, UHC Split.

Vital Parameter						
**Quantitative variables**			Average ± SD			Min–Max
Temperature [°C]			36.5 ± 0.2			36.0–36.8
Pulse [beat/minute]			86 ± 14			58–120
Systolic blood pressure [mmHg]			130 ± 26			75–170
Diastolic blood pressure [mmHg]			81 ± 13			49–100
Respiratory frequency [number/minute]			15 ± 1			14–20
Peripheral oxygen saturation [%]			97 ± 2			90–99
**Categorical variables**			N/34			
State of consciousness						
Awake			33			
Somnolence			1			
Stupor			0			
Coma			0			
Hypotension			4			
Tachycardia			8			
Dyspnoea			1			
Chest pain			0			
Changes in the electrocardiogram			0			
**Clinical records**						
Localisation of the bite			N (%)			
Upper extremity			23 (68)			
Forearm	Hand	Arm fingers	1	6	16	
Lower extremity			11 (32)			
Crus	Foot	Toes	2	5	4	
Chronic diseases (N/34)			15/34			
Cardiac and vascular disease			11			
Neurological diseases			0			
Pulmonary disease			0			
Liver diseases			0			
Mental illnesses			2			
Diabetes			5			
Malignant diseases			2			
Autoimmune disorders and/or immunosuppression			0			
Hypothyroidism			3			
Benign urological diseases			0			
Grade of envenomation			N (%)			
0			0			
1			0			
2a			24 (71)			
2b			9 (26)			
3			1 (3)			
Time from snakebite to ED—minute (median)			168			60–1140
Time from snakebite to applying antivenom—minute (median)			183			20–1170

Legend: ED—Emergency Department; Min—Minimum; Max—Maximum; SD—Standard Deviation.

**Table 2:** Clinical features and complications in *V. ammodytes*-envenomed study patients (N = 34).

**Local symptoms and signs of envenomation**	N (%)
Pain	34 (100)
Oedema	34 (100)
Redness	21 (62)
Haematoma	31 (91)
Enlarged regional lymph nodes	2 (6)
**Systemic manifestations of envenomation**	N (%)
Vomiting	11 (32)
Diarrhoea	3 (9)
Changes in mental status	1 (3)
State of shock	1 (3)
**Local complications of envenomation—N/34 (%)**	1/34 (3)
Haemorrhagic blister	1
Compartment syndrome	0
Necrosis of the skin and/or muscle	0
Thrombosis and/or thrombophlebitis	0
Infections	0
**Systemic complications of envenomation—N/34 (%)**	24/34 (71)
Paresis or paralysis of cranial nerves	8
Disorder of the state of consciousness	1
Shock	1
Clinically manifested bleeding disorder	0
Laboratory-confirmed coagulation disorder	16
Acute kidney disorder	0
Acute liver damage	0
Acute myocardial injury	0
Acute respiratory failure	0
Rhabdomyolysis and/or myositis	6
Anaemia	0
Thrombocytopenia	9
Leucocytosis	8

**Table 3:** Laboratory parameters of *V. ammodytes*-envenomed study patients (N = 34).

Laboratory Parameter	Reference Interval	Average ± SD	Min–Max
White blood cell count (×10^9^/L)	3.4–9.7 × 10^9^/L	9.9 ± 5.1	4.6–23.1
Neutrophil ratio	44–72%	67.6 ± 15.2	41.0–93.2
Red blood cell count (×10^12^/L)	4.3–5.7 × 10^12^/L	4.9 ± 0.5	4.1–6.2
Haematocrit	0.415–0.530 L/L	0.430 ± 0.067	0.154–0.558
Haemoglobin concentration	120–175 g/L	148 ± 17	120–189
Platelet count (×10^9^/L)	158–424 × 10^9^/L	210 ± 86	48–340
C-reactive protein concentration	<5.0 mg/L	3.6 ± 1.2	0.6–39.9
Glucose concentration	4.4–6.4 mmol/L	7.2 ± 3.1	4.6–19.2
Blood urea nitrogen concentration	2.8–8.3 mmol/L	6.9 ± 1.9	3.2–11.6
Serum creatinine concentration	64–104 µmol/L	81 ± 20	31–127
Sodium concentration	137–146 mmol/L	140 ± 3	134–144
Potassium concentration	3.9–5.1 mmol/L	4.0 ± 0.6	2.9–5.8
Chloride concentration	97–108 mmol/L	103 ± 2	99–108
Aspartate aminotransferase activity	11–38 U/L	31 ± 14	17–99
Alanine aminotransferase activity	12–48 U/L	26 ± 17	9–107
ɣ-glutamyl transferase activity	11–55 U/L	38 ± 22	8–147
Total bilirubin concentration	3–20 µmol/L	8 ± 4	2–15
Lactate dehydrogenase activity	25–241 U/L	192 ± 64	87–420
Creatine kinase activity	50–177 U/L	215 ± 14	42–657
High sensitive-troponin T concentration	<14 ng/L	12.7 ± 8.9	3.0–41.4
NT-proBNP concentration	<879 pg/mL	280 ± 24	10–762
Prothrombin time	>0.70	0.97 ± 0.20	0.46–1.31
Activated partial thromboplastin time	21.6–28.7 s	22.7 ± 3.0	17.3–28.6
D-dimers concentration	<0.50 mg/L	1.79 ± 0.59	0.17–11.7
Plasma fibrinogen concentration	2.0–4.0 g/L	3.30 ± 0.83	2.4–4.3

Legend: Min—Minimum; Max—Maximum; NT-proBNP—N-terminal pro B-type natriuretic peptide; SD—Standard Deviation.

**Table 4:** Treatment data and clinical outcomes in *V. ammodytes*-envenomed study patients (N = 34).

Treatment Data							
**Pre-hospital treatment**		N (%)					
Placing a tourniquet		0					
Limb immobilisation		3 (9)					
Receiving antivenom		3 (9)					
Applying supportive therapy		24 (71)					
**Hospital treatment**							
Receiving antivenom		34 (100) *					
Envenomation grade		Type of antivenom					
		Viperfav^®^ (N = 16)		BulBio^®^ (N = 17)		Viekvin^®^ (N = 1)	
2a (N = 24)		10		13		1	
1 dose	2 doses	10	0	13	0	1	0
2b (N = 9)		6		3		0	
1 dose	2 doses	4	2	1	2	/	/
3 (N = 1)		0		1		0	
1 dose	2 doses	/	/	0	1	/	/
Route of applying antivenom		Viperfav^®^ (N = 16)		BulBio^®^ (N = 17)		Viekvin^®^ (N = 1)	
IV (N = 16)		16		/		/	
IM (N = 9)		/		8		1	
IM & SC (N = 9)		/		9		/	
SC (N = 0)		/		/		/	
Supportive therapy		N (%)					
Antibiotics		18 (53)					
Human tetanus immunoglobulins		33 (97)					
Tetanus vaccine		33 (97)					
Antihistamines		31 (91)					
Corticosteroids		31 (91)					
Oxygen therapy		1 (3)					
Platelets and/or erythrocyte transfusion		0					
Anticoagulants		0					
Vasopressors		0					
Mechanical ventilation		0					
Surgical intervention		1 (3)					
Incision of haemorrhagic blister		1					
Fasciotomy		0					
Necrectomy		0					
Limb amputation		0					
Side-effects after receiving antivenom							
Early		0					
Late		0					
**Clinical outcomes**		Average ± SD					
Length of hospital stay—days		3.97 ± 1.41					
Mortality		0					

* All study patients received antivenom therapy. One patient received antivenom in the field and did not require a second dose during hospital treatment. Legend: IM—intramuscularly; IM & SC—intramuscularly and subcutaneously; SC—subcutaneously; SD—Standard Deviation.

## Data Availability

The data presented in this study are available on request from the corresponding author.

## Abbreviations

The following abbreviations are used in this manuscript:

ALT	Alanine aminotransferase
aPTT	Activated partial thromboplastin time
AST	Aspartate aminotransferase
BUN	Blood urea nitrogen
CK	Creatine kinase
Cl	Chloride
CRP	C-reactive protein
ECG	Electrocardiogram
ED	Emergency Department
ELISA	Enzyme-Linked Immunosorbent Assay
GGT	ɣ-glutamyl transferase
Hgb	Haemoglobin
hs-TnT	High sensitive-troponin T
Htc	Haematocrit
HTIG	Human tetanus immunoglobulins
ICU	Intensive Care Unit
IM	Intramuscular
IM & SC	Intramuscular and subcutaneous
INR	International Normalized Ratio
IV	Intravenous
K	Potassium
LD50	Lethal dose, 50%
LDH	Lactate dehydrogenase
Max	Maximum
Min	Minimum
mL	Milliliters
Na	Sodium
NT-proBNP	N-terminal pro B-type natriuretic peptide
PT	Prothrombin time
Ptc	Platelets
RBC	Red blood cell
RR	Riva-Rocci (comes from the name of the person inventing the non-invasive measurement of blood pressure, and refers to blood pressure measurements taken using a traditional mercury sphygmomanometer)
SC	Subcutaneous
SD	Standard deviation
TT	Tetanus toxoid (tetanus vaccine)
UHC	University Hospital Centre
WBC	White blood cell
